# Risk factors and clinical characteristics of pernicious placenta accreta in pregnancies complicated by placenta previa

**DOI:** 10.1097/MD.0000000000047453

**Published:** 2026-02-06

**Authors:** Yuan Ma, Shufen Ning, Congying Qin, Zhihui Song

**Affiliations:** a Obstetrics Department, Tangshan Maternal and Child Health Hospital, Tangshan, Hebei, China.

**Keywords:** cesarean delivery, magnetic resonance imaging, maternal outcomes, pernicious placenta previa, placenta accreta spectrum, risk factors, ultrasonography

## Abstract

This retrospective study investigated risk factors and clinical outcomes of placenta accreta in pregnancies complicated by pernicious placenta previa (PPP). A total of 177 women diagnosed with PPP and treated at our institution between July 2020 and July 2025 were included. Clinical, imaging, and perinatal data were retrieved and analyzed. Placenta accreta spectrum was diagnosed based on prenatal ultrasonography, magnetic resonance imaging, intraoperative findings, and histopathological confirmation. Univariate and multivariate logistic regression analyses were performed to identify independent predictors. Among all patients, 56 had placenta accreta and 121 did not. Women with accreta had higher rates of 2 or more prior cesarean sections, shorter intervals since the last cesarean, and anterior placental implantation (all *P* < .01). Imaging indicators, including loss of the retroplacental clear space on ultrasound and bladder wall interruption on magnetic resonance imaging, were also independently associated with accreta. Multivariate analysis identified 5 independent predictors: ≥2 previous cesarean sections (odds ratio [OR] = 5.31), shorter interpregnancy interval (OR = 0.62), anterior placental location (OR = 3.90), loss of retroplacental clear space (OR = 3.16), and bladder wall interruption (OR = 4.91). Maternal and neonatal outcomes worsened with increasing placenta accreta spectrum severity, characterized by greater intraoperative blood loss, higher transfusion and hysterectomy rates, earlier delivery, and lower neonatal birth weight and Apgar scores. Early identification of high-risk PPP patients using clinical and imaging markers, coupled with multidisciplinary management, is essential to improve maternal and neonatal outcomes.

## 1. Introduction

Placenta accreta spectrum (PAS) has emerged as a leading cause of peripartum hemorrhage and peripartum hysterectomy worldwide, with incidence rising in parallel with cesarean delivery (CD) rates. Contemporary epidemiologic analyses continue to document upward trends in PAS and related surgical morbidity, underscoring the need for precise antenatal risk stratification and standardized multidisciplinary care pathways. In pernicious placenta previa (PPP), placenta previa overlying a prior cesarean scar, the pathophysiology of abnormal placentation is thought to reflect defective decidualization and myometrial fibrosis at the scar niche, facilitating abnormal trophoblastic invasion.^[1,2]^ Clinically, PPP constitutes a concentrated reservoir of PAS risk in which both scar burden and placental topography converge. Risk profiling in PPP has evolved from simple counts of prior CDs to more nuanced constructs that incorporate the interdelivery interval and placental location. Short interpregnancy intervals after CD are increasingly recognized as markers of inadequate scar remodeling and have been linked to higher odds of PAS and related adverse outcomes; this association has been reproduced in multicenter and population-based datasets.^[3,4]^ Placental topography is also informative: anterior implantation over the cesarean scar confers substantially greater risk than posterior implantation, consistent with mechanical thinning and fibrosis at the lower uterine segment.^[5]^

Imaging is central to diagnosis and surgical planning. Ultrasound remains the first-line modality, with key signs – including loss of the retroplacental clear zone, multiple placental lacunae, subplacental hypervascularity, and myometrial thinning – showing high diagnostic accuracy in women with placenta previa and prior CDs. Recent meta-analyses and methodologic reviews from the past 3 years reaffirm robust performance for expert sonography and advocate structured scoring systems to standardize reporting.^[6]^ Magnetic resonance imaging (MRI) complements ultrasound by delineating extrauterine extension and vesical involvement; contemporary studies highlight bladder wall interruption, uterovesical hypervascularity, and loss of characteristic interfaces as particularly predictive features.^[7]^ Combined ultrasound–MRI prediction models are an active area of development and have shown incremental gains in discriminating invasive disease and guiding operative strategy.^[8]^ Despite these advances, important gaps persist. Much of the evidence intermixes heterogeneous obstetric populations, whereas PPP represents a distinct, high-risk phenotype in which scar metrics, placental topography, and imaging markers may interact differently than in general placenta previa cohorts. Moreover, relatively few studies have quantified the joint contributions of ≥ 2 prior CDs, the CD-to-conception interval, and anterior placentation after adjusting for correlated imaging signs, while simultaneously linking these determinants to graded maternal and neonatal outcomes across PAS subtypes (accreta, increta, and percreta). Recent guideline reviews call for context-specific risk models that integrate clinical and imaging domains and for outcome data that can inform timing of delivery, blood product preparation, and the threshold for cesarean hysterectomy with placenta left in situ.^[9,10]^ The innovation of this study lies in 3 aspects: it focuses on a clinically homogeneous PPP population, enabling precise evaluation of risk factors that may differ from general placenta previa cohorts; it concurrently examines scar-related factors, interpregnancy interval, placental topography, and multimodal imaging markers within a single adjusted model; and it uniquely links these predictors to stepwise maternal and neonatal outcomes across PAS severity levels, providing a more integrated risk stratification strategy.

This study aimed to investigate the clinical characteristics and risk factors associated with placenta accreta in pregnancies complicated by PPP. By analyzing maternal history, placental features, and imaging findings, the study sought to clarify the relationship between prior uterine surgery and abnormal placental invasion, and to evaluate maternal and neonatal outcomes across different degrees of placental invasion. The ultimate goal was to provide evidence-based insights to improve prenatal diagnosis, risk assessment, and perinatal management in high-risk pregnancies complicated by PPP.

## 2. Methods

### 2.1. Study design

This study was approved by the Ethics Committee of Tangshan Maternal and Child Health Hospital. This retrospective study analyzed pregnant women diagnosed with PPP who were treated at our institution between July 1, 2020, and July 1, 2025. Eligible participants were those who met the following criteria: diagnosis of PPP, defined as placenta previa occurring in women with a history of at least one cesarean section (CS) and with placental implantation overlying or adjacent to the uterine scar, confirmed by prenatal ultrasonography or MRI; singleton pregnancy with a gestational age of ≥ 28 weeks at delivery; and availability of complete clinical, imaging, and surgical records, including obstetric history, prenatal imaging findings, intraoperative details, and postoperative outcomes. Exclusion criteria included multiple pregnancies; fetal malformations or chromosomal abnormalities; placenta previa without involvement of a previous cesarean scar; concurrent uterine pathologies that could influence placental implantation, such as uterine malformations, adenomyosis, extensive myomectomy, or intrauterine adhesions; termination of pregnancy before 28 weeks of gestation; vaginal delivery; and severe systemic disorders (e.g., coagulation abnormalities, autoimmune diseases, or major cardiovascular or hepatic dysfunction) that could confound study outcomes. The study was conducted in accordance with the principles outlined in the Declaration of Helsinki and was approved by the Institutional Medical Ethics Committee. Written informed consent was obtained from all participants prior to inclusion.

### 2.2. Diagnostic criteria for PAS

The diagnosis of PAS was established based on a combination of prenatal imaging findings, intraoperative observations, and histopathological confirmation.

Prenatally, PAS was suspected when ultrasonography or MRI demonstrated characteristic features, including: loss or irregularity of the hypoechoic retroplacental zone; thinning or disruption of the uterine–bladder interface; presence of multiple placental lacunae with turbulent blood flow; abnormal hypervascularity at the uterine serosa–bladder junction on color Doppler imaging; and placental tissue extending beyond the uterine serosa or invading adjacent organs on MRI.

Intraoperatively, PAS was diagnosed when 1 or more of the following features were observed: failure of placental separation after delivery of the fetus despite routine management; abnormally firm adherence of the placenta to the myometrium; diffuse bleeding from the placental bed; and visible placental tissue invading the myometrium, serosa, or adjacent pelvic organs.

Definitive diagnosis was confirmed by postoperative histopathological examination, which demonstrated chorionic villi abnormally adherent to, penetrating, or perforating the myometrium, corresponding respectively to placenta accreta, increta, or percreta.

### 2.3. Data collection

Clinical data of all patients diagnosed with PPP were retrospectively collected from the electronic medical record system of our hospital between July 1, 2020, and July 1, 2025. Two experienced obstetricians independently reviewed and verified the medical records to ensure data accuracy and completeness.

The collected variables included: demographic data: maternal age, body mass index (BMI), gravidity, parity, and gestational age at diagnosis. Obstetric history: number of prior CSs, history of uterine curettage, interval between the last CS and the current pregnancy, and previous placenta previa. Prenatal imaging data: placental location, placental thickness, and ultrasound or MRI findings related to PAS, including loss of the retroplacental clear space, presence of multiple placental lacunae, hypervascularity at the uterine–bladder interface, and bladder wall interruption. Laboratory indicators: serum alpha-fetoprotein (AFP) level and routine biochemical results obtained during prenatal assessment. Intraoperative and perinatal outcomes: intraoperative blood loss, requirement for blood transfusion, hysterectomy, gestational age at delivery, neonatal birth weight, 1-minute Apgar score, and neonatal intensive care unit admission.

Data were cross-checked by 2 investigators, and discrepancies were resolved by consensus with a senior attending obstetrician. Patients with missing key clinical or imaging information were excluded.

### 2.4. Statistical analysis

All statistical analyses were performed using Statistical Package for the Social Sciences version 27.0 (IBM Corp., Armonk) and R version 4.4.1 (R Foundation for Statistical Computing, Vienna, Austria). Continuous variables were tested for normality using the Shapiro–Wilk test. Normally distributed data were expressed as mean ± standard deviation, while non-normally distributed data were presented as median and interquartile range. Categorical variables were expressed as frequencies and percentages (n, %). Comparisons between the placenta accreta and non-accreta groups were conducted using the independent-samples *t*-test or Welch *t*-test for continuous variables with normal distribution, and the Mann–Whitney *U* test for non-normally distributed variables. Categorical variables were compared using the Pearson chi-square test or Fisher exact test, as appropriate. Variables with *P* < .10 in univariate analysis, as well as those considered clinically relevant, were included in a multivariate logistic regression model to identify independent risk factors for placenta accreta. The results were reported as odds ratios (ORs) with 95% confidence intervals (CIs). Model fit was evaluated using the Hosmer–Lemeshow goodness-of-fit test, and explanatory power was assessed with the Nagelkerke *R*^2^ value. The overall predictive accuracy was expressed as the correct classification rate. Multicollinearity among independent variables was assessed using tolerance and the variance inflation factor (VIF), with VIF < 10 considered acceptable. For variables with multiple subgroup levels, such as different grades of PAS (accreta, increta, and percreta), comparisons of pregnancy outcomes were performed using one-way analysis of variance or the Kruskal–Wallis test. All statistical tests were two-tailed, and a *P* value < .05 was considered statistically significant.

## 3. Results

### 3.1. Baseline characteristics of the study population

A total of 177 women diagnosed with PPP were included in this study, comprising 56 patients with placenta accreta and 121 without accreta. The mean maternal age of the entire cohort was 33.9 ± 4.5 years, and the mean BMI was 24.8 ± 3.2 kg/m^2^. The mean gravidity and parity were 3.6 ± 1.3 and 1.9 ± 0.7, respectively. Regarding obstetric history, 32 women (18.1%) had 2 or more prior CSs, and 107 (60.5%) had a history of uterine curettage. Thirty (16.9%) patients had a previous episode of placenta previa. The mean interval from the last CS to the current pregnancy was 4.4 ± 2.1 years.

Placental characteristics based on prenatal imaging showed that 107 patients (60.5%) had anterior placental implantation, while 70 (39.5%) had posterior implantation. Ultrasound findings indicative of abnormal placental attachment were observed in a notable proportion of cases, including loss of the retroplacental clear space in 88 patients (49.7%), multiple placental lacunae in 82 (46.3%), and hypervascularity at the uterine–bladder interface in 74 (41.8%). On MRI, 24 patients (13.6%) exhibited evidence of bladder wall thinning or interruption. The mean placental thickness was 42.5 ± 7.7 mm, and the mean gestational age at diagnosis was 30.3 ± 2.5 weeks. The mean serum AFP level was 102 ± 26 ng/mL. Thirteen patients (7.3%) conceived through in vitro fertilization (IVF), and 8 (4.5%) reported smoking during pregnancy.

### 3.2. Univariate analysis of risk factors for placenta accreta in patients with PPP

Univariate analysis was performed to identify potential factors associated with placenta accreta in patients with PPP. As shown in Table 1, patients with placenta accreta had a significantly higher proportion of those with 2 or more previous cesarean deliveries compared with those without accreta (35.7% vs 9.9%, *P* < .001). The interval between the last CS and the current pregnancy was markedly shorter in the accreta group (3.3 ± 1.8 years vs 4.9 ± 2.2 years, *P* < .001). Anterior placental implantation was more frequently observed among patients with accreta (82.1% vs 50.4%, *P* < .001). Characteristic imaging findings were also significantly associated with the presence of placenta accreta. Specifically, the accreta group showed higher incidences of loss of the retroplacental clear space on ultrasound (73.2% vs 38.8%, *P* < .001), multiple placental lacunae (71.4% vs 34.7%, *P* < .001), hypervascularity at the uterine–bladder interface on Doppler examination (67.9% vs 29.8%, *P* < .001), and bladder wall interruption on MRI (28.6% vs 6.6%, *P* < .001). No statistically significant differences were found between the 2 groups in maternal age, BMI, gravidity, parity, history of uterine curettage, previous placenta previa, placental thickness, gestational age at diagnosis, or serum AFP levels (all *P* > .05). Likewise, rates of IVF conception and smoking during pregnancy did not differ significantly between groups (*P* = .553 and *P* = .709, respectively).

**Table 1:** Univariate analysis of factors associated with placenta accreta among pernicious placenta previa.

Variable	Placenta accreta (n = 56)	Non-accreta (n = 121)	Statistic	*P*-value
Maternal age, yr	34.4 ± 4.1	33.6 ± 4.7	1.150	.252
BMI, kg/m^2^	25.0 ± 3.2	24.6 ± 3.1	0.780	.437
Gravidity, times	3.9 ± 1.3	3.5 ± 1.2	1.950	.054
Parity, times	2.0 ± 0.8	1.8 ± 0.7	1.610	.111
Prior cesareans ≥ 2, n/N (%)	20/56 (35.7)	12/121 (9.9)	17.200	<.001
Uterine curettage history, n/N (%)	39/56 (69.6)	68/121 (56.2)	2.890	.089
Prior placenta previa, n/N (%)	12/56 (21.4)	18/121 (14.9)	1.170	.280
Interval from last CS, yr	3.3 ± 1.8	4.9 ± 2.2	−5.110	<.001
Anterior placental location, n/N (%)	46/56 (82.1)	61/121 (50.4)	16.120	<.001
Placental thickness, mm	43.8 ± 7.9	41.9 ± 7.6	1.510	.135
Loss of retroplacental clear space (US), n/N (%)	41/56 (73.2)	47/121 (38.8)	18.090	<.001
Multiple placental lacunae (US), n/N (%)	40/56 (71.4)	42/121 (34.7)	20.760	<.001
Hypervascularity at uterine–bladder interface (Doppler), n/N (%)	38/56 (67.9)	36/121 (29.8)	22.850	<.001
Bladder wall interruption (MRI), n/N (%)	16/56 (28.6)	8/121 (6.6)	15.750	<.001
Gestational age at diagnosis, wk	30.1 ± 2.5	30.5 ± 2.4	−1.000	.319
Serum AFP, ng/mL	105 ± 26	101 ± 25	0.960	.338
IVF conception, n/N (%)	5/56 (8.9)	8/121 (6.6)	0.302	.583
Smoking during pregnancy, n/N (%)	3/56 (5.4)	5/121 (4.1)	0.133	.715

AFP = alpha-fetoprotein, BMI = body mass index, CS = cesarean section, IVF = in vitro fertilization, MRI = magnetic resonance imaging, US = ultrasound.

### 3.3. Multivariate logistic regression analysis of risk factors for placenta accreta in patients with PPP

Variables that showed statistical significance in univariate analysis were included in a multivariate logistic regression model. As shown in Table 2, 5 factors were independently associated with placenta accreta in patients with PPP. A history of 2 or more previous cesarean deliveries was strongly correlated with placenta accreta (OR = 5.31, 95% CI: 1.85–15.23, *P* = .002). A shorter interval since the last CS was also identified as an independent risk factor (OR = 0.62, 95% CI: 0.45–0.85, *P* = .003). In addition, anterior placental location significantly increased the risk (OR = 3.90, 95% CI: 1.45–10.49, *P* = .007). Among imaging findings, both loss of the retroplacental clear space on ultrasound (OR = 3.16, 95% CI: 1.26–7.94, *P* = .014) and bladder wall interruption on MRI (OR = 4.91, 95% CI: 1.43–16.81, *P* = .011) were identified as independent predictors. Although multiple placental lacunae and hypervascularity at the uterine–bladder interface demonstrated positive associations, they did not reach statistical significance after adjustment (*P* = .056 and *P* = .058, respectively).

**Table 2:** Multivariate logistic regression analysis of independent risk factors for placenta accreta in patients with pernicious placenta previa.

Variable	β	SE	Wald χ^2^	OR (95% CI)	*P*-value
Prior cesareans ≥ 2	1.670	0.540	9.570	5.31 (1.85–15.23)	.002
Interval from last CS, yr	−0.480	0.160	9.000	0.62 (0.45–0.85)	.003
Anterior placental location	1.360	0.500	7.400	3.90 (1.45–10.49)	.007
Loss of retroplacental clear space (US)	1.150	0.470	6.000	3.16 (1.26–7.94)	.014
Multiple placental lacunae (US)	0.880	0.460	3.665	2.41 (0.98–5.94)	.056
Hypervascularity at uterine–bladder interface (Doppler)	0.910	0.480	3.601	2.48 (0.97–6.30)	.058
Bladder wall interruption (MRI)	1.590	0.630	6.390	4.91 (1.43–16.81)	.011
Constant	−5.020	1.180	18.100	–	<.001

CI = confidence interval, CS = cesarean section, MRI = magnetic resonance imaging, OR = odds ratio, SE = standard error, US = ultrasound.

### 3.4. Multicollinearity diagnostics

To evaluate potential multicollinearity among variables entered into the multivariate logistic regression model, collinearity diagnostics were performed using the tolerance and VIF. As shown in Table 3, all tolerance values were above 0.5 and all VIF values were below 2.0, indicating an acceptable level of collinearity among the included predictors. Specifically, the variables “prior cesareans ≥ 2” and “interval from last CS” demonstrated mild correlation (VIF = 1.63 and 1.71, respectively), which was expected due to shared obstetric history, but these values were well below the commonly accepted threshold of 10.0 for significant multicollinearity. Similarly, imaging-related predictors – including “loss of retroplacental clear space,” “multiple placental lacunae,” “hypervascularity at the uterine–bladder interface,” and “bladder wall interruption on MRI” – showed VIF values between 1.28 and 1.65, confirming the independence of imaging parameters in the model.

**Table 3:** Multicollinearity diagnostics for variables included in the multivariate logistic regression model.

Variable	Tolerance	VIF
Prior cesareans ≥ 2	0.612	1.634
Interval from last CS, yr	0.584	1.713
Anterior placental location	0.667	1.499
Loss of retroplacental clear space (US)	0.714	1.400
Multiple placental lacunae (US)	0.658	1.520
Hypervascularity at uterine–bladder interface (Doppler)	0.606	1.650
Bladder wall interruption (MRI)	0.782	1.279

CS = cesarean section, MRI = magnetic resonance imaging, US = ultrasound, VIF = variance inflation factor.

### 3.5. Comparison of pregnancy outcomes by placenta type and degree of PAS

Across placenta types and degrees of PAS, maternal and neonatal outcomes differed significantly. Mean gestational age at delivery decreased with deeper invasion (non-accreta 36.3 ± 1.9 weeks; accreta 35.4 ± 2.1; increta 34.9 ± 2.0; percreta 34.5 ± 2.3; one-way analysis of variance F = 5.520, *P* = .001). Consistently, the rate of preterm delivery (< 37 weeks) increased across groups (40.5%, 67.9%, 77.8%, and 80.0%, respectively; χ^2^ = 17.053, *P* = .001). Intraoperative blood loss rose stepwise from non-accreta to percreta (median [interquartile range]: 950 [600–1500] mL; 1600 [1200–2200] mL; 2400 [1600–3200] mL; 3400 [2500–4600] mL; Kruskal–Wallis, *P* < .001). Parallel gradients were observed for transfusion requirement (34.7%, 75.0%, 94.4%, 100.0%; χ^2^ = 42.498, *P* < .001) and hysterectomy (1.7%, 14.3%, 33.3%, 70.0%; χ^2^ = 57.032, *P* < .001). Neonatal outcomes showed similar trends. Mean birth weight declined with increasing invasion depth (2870 ± 470 g; 2640 ± 520 g; 2480 ± 490 g; 2380 ± 560 g; F = 6.679, *P* < .001). The proportion with 1-minute Apgar score < 7 increased (7.4%, 17.9%, 33.3%, 40.0%; χ^2^ = 16.279, *P* = .001), as did neonatal intensive care unit admissions (9.9%, 25.0%, 33.3%, 40.0%; χ^2^ = 13.056, *P* = .005; Table 4).

**Table 4:** Comparison of pregnancy outcomes among placenta types and PAS grades.

Outcome variable	Placenta previa without accreta (n = 121)	Placenta accreta (n = 28)	Placenta increta (n = 18)	Placenta percreta (n = 10)
Gestational age at delivery (wk)	36.3 ± 1.9	35.4 ± 2.1	34.9 ± 2.0	34.5 ± 2.3
Preterm delivery (<37 wk), n (%)	49 (40.5)	19 (67.9)	14 (77.8)	8 (80.0)
Intraoperative blood loss (mL)	950 [600–1500]	1600 [1200–2200]	2400 [1600–3200]	3400 [2500–4600]
Blood transfusion required, n (%)	42 (34.7)	21 (75.0)	17 (94.4)	10 (100.0)
Hysterectomy performed, n (%)	2 (1.7)	4 (14.3)	6 (33.3)	7 (70.0)
Neonatal birth weight (g)	2870 ± 470	2640 ± 520	2480 ± 490	2380 ± 560
Apgar score < 7 at 1 min, n (%)	9 (7.4)	5 (17.9)	6 (33.3)	4 (40.0)
NICU admission, n (%)	12 (9.9)	7 (25.0)	6 (33.3)	4 (40.0)

NICU = neonatal intensive care unit, PAS = placenta accreta spectrum.

## 4. Discussion

This study examined risk factors and clinical characteristics of placenta accreta in pregnancies complicated by PPP and evaluated maternal–neonatal outcomes across the PAS. Several observations emerge. First, among women with PPP, a history of ≥ 2 cesarean deliveries (CDs), a shorter interval since the last CD, anterior placental implantation, loss of the retroplacental clear space on ultrasound, and bladder wall interruption on MRI were independently associated with placenta accreta. Second, maternal and neonatal outcomes showed a stepwise deterioration from non-accreta to accreta, increta, and percreta, with increasing hemorrhage, transfusion, and cesarean hysterectomy, and with earlier delivery, lower birth weight, and higher neonatal morbidity. Third, other baseline factors – including maternal age, BMI, gravidity, parity, serum AFP, IVF conception, and smoking – were not independently associated with accreta in this PPP cohort.

The association between cumulative uterine scar burden and PAS risk is biologically plausible and consistent with the scar-implantation model of abnormal trophoblast–myometrium interface. In our cohort, ≥2 prior CDs increased the odds of accreta more than fivefold. This finding aligns with contemporary large-scale clinical datasets showing a monotonic increase in PAS prevalence with the number of CDs, especially in the presence of placenta previa or low-lying placenta, and higher odds when the placenta is anterior over a scar.^[11]^ The shorter interval since the last CD independently lowered the OR per year, supporting the hypothesis that inadequate scar remodeling increases implantation vulnerability; recent population-based and cohort analyses similarly report elevated PAS risk with short interpregnancy or interdelivery intervals after CD.^[12]^

An anterior placental location remained an independent predictor after adjustment. This is consistent with reports that anterior previa over a scar confers higher PAS odds compared with posterior implantation, likely because the placenta overlies the thinnest portion of the lower uterine segment and the prior hysterotomy site. Contemporary analyses confirm the interaction between anterior location and increasing CD number in driving PAS risk.^[13]^ Ultrasound markers contributed meaningful discriminative information. Loss of the retroplacental clear space, reflecting disruption of the decidua basalis and myometrial–placental interface, remained independently associated with accreta. Multiple lacunae and hypervascularity showed strong univariate associations but did not retain significance after adjustment, suggesting collinearity among imaging signs and with clinical risk factors. Recent studies have refined ultrasound scoring systems in which lacunae, clear zone loss, and subplacental hypervascularity are dominant predictors and demonstrate good to excellent diagnostic performance across trimesters.^[14]^ MRI features also provided incremental value; bladder wall interruption remained an independent predictor, consistent with the literature that bladder wall irregularity, tenting, and abnormal vessels near the serosa are among the most accurate MRI signs of parametrial or vesical invasion.^[15]^ Combined ultrasound–MRI strategies have been shown in recent work to improve diagnostic accuracy and prognostication for invasive PAS and related adverse outcomes, supporting a multimodal imaging pathway for high-risk PPP.^[16,17]^

Outcome analyses demonstrated a graded increase in surgical complexity and morbidity with deeper invasion. Blood loss, transfusion, and hysterectomy increased from accreta to increta to percreta, mirroring current consensus that percreta carries the highest risk of massive hemorrhage and adjacent organ injury. Our results corroborate guideline-based management algorithms that recommend planned delivery in a level III/IV center with a multidisciplinary team, blood bank preparation, and avoidance of forcible placental removal; cesarean hysterectomy with the placenta left in situ remains the most widely accepted approach for definitive management.

The absence of independent associations for several baseline variables deserves comment. Maternal age, BMI, gravidity, and parity were not significant after adjustment. Recent multicenter reports variably identify BMI ≥ 30 kg/m^2^ and other uterine surgeries as contributors, but the effect sizes are smaller than those of scar-related variables and may attenuate once scar number, scar interval, and placental topography are included. IVF conception and smoking were also not associated in our PPP cohort; literature is mixed, with some analyses suggesting small effects for IVF or smoking in broader obstetric populations, but these effects appear to be overshadowed by scar-placenta interactions in PPP.^[18]^ Our multicollinearity diagnostics indicated acceptable independence among predictors, with all VIFs < 2.0. Mild correlation between “≥2 prior CDs” and “scar interval” is expected, yet values remained far below thresholds associated with unstable regression estimates, supporting the robustness of the adjusted associations. This methodological observation is pertinent because multiple imaging features are interrelated and often co-occur with anterior previa; nonetheless, the retained independent predictors (anterior location, clear space loss, and bladder wall interruption) are mechanistically coherent and reflective of pathologic invasion.^[19]^

The present findings are congruent with and extend recent work published within the last 3 years. A large contemporary analysis reported that PAS rates increase sharply with the number of prior CDs and are highest when placenta previa or anterior low-lying placenta overlies the scar; ORs escalated with each additional CD, paralleling our observation that ≥ 2 CDs markedly increased accreta risk in PPP. With respect to interpregnancy timing, cohort and population-based studies from 2022 to 2024 reported that short intervals after CD were associated with PAS or with the composite of PAS/placenta previa, likely reflecting inadequate scar remodeling; our adjusted model identifying shorter scar interval as an independent predictor aligns with this evidence.

Regarding imaging, a 2023 to 2025 body of research refined ultrasound-based scoring systems and trimester-specific performance, emphasizing lacunae, loss of the clear zone, myometrial thinning, and subplacental hypervascularity as core signs, with high diagnostic accuracy when assessed by experienced operators.^[14]^ Our findings accord with this framework but show that, after multivariable adjustment, clear zone loss retained significance whereas lacunae and hypervascularity did not, likely due to intercorrelation among signs and with anterior placentation. On MRI, recent systematic reviews and practice-oriented reviews highlight bladder wall interruption and tenting as among the most predictive features of vesical involvement and advanced invasion, consistent with our independent association for bladder wall interruption.^[20]^ Finally, management recommendations continue to favor planned cesarean hysterectomy with the placenta left in situ, multidisciplinary planning, and delivery in centers with PAS expertise; our outcome gradients (hemorrhage, transfusion, and hysterectomy) across PAS severity underscore the clinical rationale behind these guidelines. Collectively, our results are directionally consistent with recent literature but add granularity by jointly quantifying scar number, scar interval, placental topography, and specific imaging markers within a single PPP cohort, while demonstrating dose–response in outcomes across PAS subtypes. Compared with recent studies, the strength of our dataset lies in its homogeneity, as all patients had PPP overlying a previous cesarean scar. Additionally, both ultrasound and MRI findings were incorporated into a single adjusted model, allowing identification of truly independent imaging predictors. The observation that interpregnancy interval and anterior placentation remained independent factors provides new evidence that structural scar remodeling and placental topography interact uniquely in PPP. Furthermore, this study provides detailed graded outcome analyses across PAS severity, offering new evidence for clinical decision-making.

These data support a structured risk-stratification pathway in PPP that concurrently considers scar burden (≥2 CDs), scar interval, anterior placentation, and targeted imaging markers (clear zone loss on ultrasound and bladder wall interruption on MRI). In practice, PPP patients with 1 or more of these features should be triaged early to specialist centers, undergo multidisciplinary delivery planning, and receive blood product preparation. Combined ultrasound–MRI assessment can be reserved for equivocal cases or suspected deep invasion. Adopting this algorithm is likely to reduce emergent hemorrhage, shorten decision-to-incision time, and optimize neonatal timing in high-risk PPP. This study has several strengths. It focuses on a clinically homogeneous high-risk population (PPP) with standardized imaging definitions and operative/pathologic verification where available. The analysis incorporates both scar metrics (number and interval) and placental topography, reducing confounding by indication. Imaging variables were curated from sonographic and MRI domains, and multicollinearity diagnostics were explicitly reported, supporting the stability of the multivariable estimates.

For patients with multiple high-risk features (≥2 prior CSs, anterior placenta, clear space loss, or MRI-suggested bladder involvement), we recommend planned CD between 34 + 0 and 36 + 0 weeks in a level III/IV center. MRI evaluation should be performed when ultrasound findings are inconclusive or when bladder involvement is suspected. Multidisciplinary team (MDT) participation – including obstetrics, anesthesiology, neonatology, urology, interventional radiology, and blood bank – is strongly recommended for all cases with suspected PAS. Preoperative preparation should include availability of ≥ 6 units of packed RBCs and access to massive transfusion protocol.

Limitations should be acknowledged. First, the retrospective single-institution design limits external generalizability and invites selection bias. Second, although imaging was interpreted by experienced operators, interobserver variability was not formally quantified, and ultrasound performance is operator dependent. Third, the sample size within PAS subtypes, particularly percreta, was modest, which widens CIs and may underpower detection of differences for some predictors (e.g., lacunae and hypervascularity) after adjustment. Fourth, intraoperative blood loss was analyzed by group summaries; granular anesthetic and hemostatic variables (cell salvage and interventional radiology use) were not modeled and could contribute to outcome heterogeneity. Fifth, delivery timing and intent (planned vs emergent) were not included in risk models and could mediate part of the association between imaging severity and outcomes. Prospective multicenter validation with standardized imaging acquisition, blinded adjudication, and protocolized peripartum management would strengthen causal inference and facilitate generalization.

## 5. Conclusions

In pregnancies complicated by PPP, placenta accreta was strongly associated with a history of multiple cesarean deliveries, a shorter interval since the last cesarean, and anterior placental implantation. Ultrasound findings of retroplacental clear space loss and MRI evidence of bladder wall interruption were independent predictors of invasion. Increasing severity of placental adherence correlated with greater blood loss, transfusion requirement, hysterectomy, and poorer neonatal outcomes. Early identification of high-risk patients and multidisciplinary management are essential to reduce maternal and neonatal morbidity in PPP-associated PAS disorders.

## Author contributions

**Conceptualization:** Yuan Ma, Shufen Ning, Congying Qin, Zhihui Song.

**Data curation:** Yuan Ma, Shufen Ning, Congying Qin, Zhihui Song.

**Formal analysis:** Yuan Ma, Shufen Ning, Congying Qin, Zhihui Song.

**Funding acquisition:** Yuan Ma, Zhihui Song.

**Investigation:** Zhihui Song.

**Writing – original draft:** Shufen Ning, Congying Qin, Zhihui Song.

**Writing – review & editing:** Shufen Ning, Congying Qin, Zhihui Song.

## Correction

The funding information for this paper has now been updated online with the footnote statement: “This study was supported by the “Medical Science Research Project of Hebei” under Grant No. 20231744.”

## References

[1] Huang C Peng Z Li L. Application of double-row transfixion suture of the lower uterine segment in cesarean section for pernicious placenta previa complicated by placenta accreta spectrum: a comparative clinical study. BMC Pregnancy Childbirth. 2025;25:381. 40175946 10.1186/s12884-025-07469-4 PMC11963370

[2] Zhao H Wang Q Han M Xiao X. Current state of interventional procedures to treat pernicious placenta previa accompanied by placenta accreta spectrum: a review. Medicine (Baltim). 2023;102:e34770. 10.1097/MD.0000000000034770 PMC10508584 37713901

[3] Ma Y Ma J. Interpregnancy interval: attention should be given after conservative treatment of placenta accreta spectrum. Am J Obstet Gynecol MFM. 2024;6:101252. 38070677 10.1016/j.ajogmf.2023.101252

[4] Zhao H Liu C Fu H Abeykoon SDI Zhao X. Subsequent pregnancy outcomes and risk factors following conservative treatment for placenta accreta spectrum: a retrospective cohort study. Am J Obstet Gynecol MFM. 2023;5:101189. 37832645 10.1016/j.ajogmf.2023.101189

[5] Bonanni G Lopez-Giron MC Allen L. Guidelines on placenta accreta spectrum disorders: a systematic review. JAMA Netw Open. 2025;8:e2521909. 40679824 10.1001/jamanetworkopen.2025.21909 PMC12274978

[6] Maged AM El-Mazny A Kamal N. Diagnostic accuracy of ultrasound in the diagnosis of placenta accreta spectrum: systematic review and meta-analysis. BMC Pregnancy Childbirth. 2023;23:354. 37189095 10.1186/s12884-023-05675-6 PMC10186814

[7] Jariyawattanarat W Thiravit S Suvannarerg V Srisajjakul S Sutchritpongsa P. Bladder involvement in placenta accreta spectrum disorder with placenta previa: MRI findings and outcomes correlation. Eur J Radiol. 2023;160:110695. 36657210 10.1016/j.ejrad.2023.110695

[8] Kang Y Zhong Y Qian W Yue Y Peng L. A prediction model based on MRI and ultrasound to predict the risk of PAS in patient with placenta previa. Eur J Obstet Gynecol Reprod Biol. 2024;301:227–33. 39159508 10.1016/j.ejogrb.2024.08.002

[9] Zou L Wang P Song Z. Effectiveness of a fetal magnetic resonance imaging scoring system for predicting the prognosis of pernicious placenta previa: a retrospective study. Front Physiol. 2022;13:921273. 36035494 10.3389/fphys.2022.921273 PMC9402898

[10] Guo P Wu Y Yuan X Wan Z. Clinical diagnostic value and analysis of MRI combined with ultrasound in prenatal pernicious placenta previa with placenta accreta. Ann Palliat Med. 2021;10:6753–9. 34237975 10.21037/apm-21-1285

[11] White A Malik M Pruszynski JE Do QN Spong CY Herrera CL. Contemporary placenta accreta spectrum disorder incidence and risk factors. Obstet Gynecol. 2025;145:665–73. 40245410 10.1097/AOG.0000000000005919 PMC12278684

[12] McLaughlin HD Benson AE Scaglione MA. Association between short interpregnancy interval and placenta accreta spectrum. AJOG Glob Rep. 2022;2:100051. 36275493 10.1016/j.xagr.2022.100051 PMC9563919

[13] ADoPAD (Antenatal Diagnosis of Placental Attachment Disorders) Study Group. Risk factors, prenatal diagnosis, and outcome of posterior placenta accreta spectrum disorders in patients with placenta previa or low-lying placenta: a multicenter study. Acta Obstet Gynecol Scand. 2025;104:1328–38. 40387324 10.1111/aogs.15132 PMC12144582

[14] Zhang J Li H Feng D Wu J Wang Z Feng F. Ultrasound scoring system for prenatal diagnosis of placenta accreta spectrum. BMC Pregnancy Childbirth. 2023;23:569. 37550654 10.1186/s12884-023-05886-x PMC10405485

[15] Shuai X Gao C Zhang H. Bladder involvement in placenta accreta spectrum disorders: 2D US combined with the 3D crystal Vue and MRI comparative analysis. BMC Pregnancy Childbirth. 2024;24:788. 39593009 10.1186/s12884-024-06997-9 PMC11590337

[16] Zhang J Kong L Qu F. The predictive value of conventional magnetic resonance imaging combined with intravoxel incoherent motion parameters for evaluating maternal and neonatal clinical outcomes in patients with placenta accreta spectrum disorders. Placenta. 2024;151:10–7. 38631235 10.1016/j.placenta.2024.04.007

[17] Lu T Song B Pu H. Prognosticators of intravoxel incoherent motion (IVIM) MRI for adverse maternal and neonatal clinical outcomes in patients with placenta accreta spectrum disorders. Transl Androl Urol. 2020;9:258–66. 32420131 10.21037/tau.2019.12.27 PMC7214992

[18] Kayem G Seco A Vendittelli F. Risk factors for placenta accreta spectrum disorders in women with any prior cesarean and a placenta previa or low lying: a prospective population-based study. Sci Rep. 2024;14:6564. 38503816 10.1038/s41598-024-56964-9 PMC10951207

[19] You H Wang Y Han R Gu J Zeng L Zhao Y. Risk factors for placenta accreta spectrum without prior cesarean section: a case-control study in China. Int J Gynaecol Obstet. 2024;166:1092–9. 38573157 10.1002/ijgo.15493

[20] AbdelAziz S El-Goly NA Maged AM Bassiouny N El-Demiry N Shamel A. Diagnostic accuracy of magnetic resonance imaging in the diagnosis of placenta accreta spectrum: a systematic review and meta-analysis. Matern Fetal Med. 2025;7:15–21. 40620617 10.1097/FM9.0000000000000241 PMC12222980

